# Sleep Hygiene and Symptom Burden in Multiple Sclerosis: A Cross-Sectional Study

**DOI:** 10.3390/clockssleep8020024

**Published:** 2026-04-30

**Authors:** Michalina Rzepka, Aleksandra Buczek, Tomasz Chmiela, Weronika Galus, Joanna Siuda, Ewa Krzystanek

**Affiliations:** 1 Department of Neurology, Faculty of Medical Sciences in Katowice, Medical University of Silesia, 40-752 Katowice, Poland; wgalus@sum.edu.pl (W.G.); jsiuda@sum.edu.pl (J.S.); 2 Students’ Scientific Society, Department of Neurology, Faculty of Medical Sciences in Katowice, Medical University of Silesia, 40-752 Katowice, Poland; aleksandra.buczek26@gmail.com; 3 Department of Neurology, Mayo Clinic, Jacksonville, FL 32224, USA; 4 Department of Neurology, Faculty of Health Sciences in Katowice, Medical University of Silesia, 40-635 Katowice, Poland; ekrzystanek@sum.edu.pl

**Keywords:** multiple sclerosis, sleep–wake disorders, sleep hygiene, fatigue, insomnia, depression, anxiety, sleep quality

## Abstract

Sleep disturbances are common in multiple sclerosis (MS) and contribute to increased symptom burden. Behavioral sleep hygiene practices are potentially modifiable factors influencing sleep and related symptoms, yet their role in MS remains insufficiently explored. This cross-sectional study comprised 175 MS patients. Sleep hygiene was assessed using a behavioral checklist covering a regular sleep schedule, avoidance of daytime naps, limitation of evening caffeine intake, reduced evening screen exposure, and optimization of the sleep environment. The instruments included the Fatigue Severity Scale (FSS), the Modified Fatigue Impact Scale (MFIS), the Athens Insomnia Scale (AIS), the Epworth Sleepiness Scale (ESS), and the Hospital Anxiety and Depression Scale (HADS). Higher sleep hygiene adherence was associated with lower daytime sleepiness (ESS: r = −0.18, *p* = 0.020), anxiety (HADS-A: r = −0.16, *p* = 0.034), and depression (HADS-D: r = −0.15, *p* = 0.047). Patients with higher adherence (score ≥ 3) demonstrated significantly lower MFIS, AIS, ESS, and HADS-D scores compared with those with lower adherence (all *p* < 0.05). Multivariate regression showed that sleep hygiene adherence was independently associated with lower MFIS (β = −3.24, 95% CI: −6.06 to −0.41, *p* = 0.025), ESS (β = −0.85, 95% CI: −6.06 to −0.41, *p* = 0.016), HADS-A (β = −0.67, 95% CI: −1.23 to −0.11, *p* = 0.019), and HADS-D scores (β = −0.62, 95% CI: −1.17; −0.08, *p* = 0.026). Better adherence to sleep hygiene practices may be associated with a lower symptom burden in MS.

## 1. Introduction

Insufficient and disturbed sleep is prevalent among patients diagnosed with multiple sclerosis (MS) and has been demonstrated to have wide-ranging clinical consequences [1,2,3]. The etiology of sleep disturbance can be multifactorial, with potential contributors including neurological damage affecting sleep-regulating brain regions, inflammatory processes, chronic pain, spasticity, nocturia, and medication effects [4,5,6,7]. It has been established that insomnia and obstructive sleep apnea are particularly common sleep disorders in patients with MS (pwMS) [2,3]. Untreated insomnia and other sleep disorders have been shown to exacerbate fatigue, cognitive dysfunction, and mood disorders—already major burdens in MS [8]. PwMS with sleep disturbances report a significantly diminished quality of life (QoL) [9,10]. In contrast, a timely diagnosis and treatment of sleep disorders in pwMS may reduce fatigue, improve general health, and enhance QoL [11]. As these symptoms have been demonstrated to have a significant impact on QoL in pwMS, the enhancement of sleep has been identified as a key therapeutic objective [9,10,11]. Consequently, the promotion of adequate sleep hygiene should be a fundamental component of a personalized approach to the management of symptoms.

Fatigue is among the most prevalent and disabling symptoms in MS, affecting 50–90% of patients [12]. Besides disease-related factors, secondary fatigue may result from comorbidities such as sleep and mood disorders, with depression exhibiting the highest prevalence [13]. A cross-sectional study by Rzepka et al. (2024) has previously demonstrated a strong correlation between sleep disturbances and diminished physical and mental well-being in pwMS [14].

Most previous research has focused on sleep quality, insomnia severity, or specific sleep disorders [15,16,17]. However, the role of sleep hygiene in MS populations remains insufficiently explored. Behavioral sleep hygiene practices have received less attention, and standardized approaches to measuring sleep hygiene in MS populations are lacking. It constitutes a modifiable behavioral domain that may be targeted through low-cost interventions [18]. Sleep hygiene is defined as a set of behavioral and environmental practices that promote healthy sleep [19]. These practices include the maintenance of a regular sleep schedule, the avoidance of stimulants in the period prior to bedtime, the limitation of evening exposure to electronic screens, the minimization of daytime naps, and the maintenance of a comfortable sleep environment [20]. It is proposed that a conceptual model exists in which poor sleep hygiene contributes to sleep fragmentation and circadian dysregulation [18]. These, in turn, may result in increased fatigue, daytime sleepiness, and mood disturbance symptoms in pwMS [21].

Improving sleep hygiene and treating sleep disorders may therefore alleviate these comorbidities. Cognitive behavioral therapy for insomnia (CBT-I) has been proven to be an effective treatment for insomnia, depression, and fatigue [22,23,24,25]. In MS, clinical guidelines emphasize the importance of sleep management and hygiene [12,13].

Despite its clinical relevance, quantitative studies examining the relationship between sleep hygiene behaviors and symptom burden in MS remain limited [26,27]. The objective of this study was to evaluate the association between sleep hygiene adherence and symptom burden in a cohort of pwMS who have been clinically characterized. The hypothesis was formulated that greater adherence to behaviors conducive to sleep would be associated with reduced fatigue, insomnia severity, daytime sleepiness, anxiety, and depression.

## 2. Results

### 2.1. Baseline Characteristics of the Study Population

The study cohort consisted of 175 pwMS, predominantly female (74.9%) and with a mean age of 38.8 years. Most participants had been diagnosed with relapsing–remitting MS (89.7%), with mild-to-moderate disability (median Expanded Disability Status Scale (EDSS) 2.0), and were receiving disease-modifying therapy (94.3%). Key demographic and clinical characteristics are presented in Table 1.

### 2.2. Impact of Sleep Hygiene Adherence on Symptoms

The sleep hygiene adherence score ranged from 0 to 5 (mean 2.85 ± 1.02). Item-level adherence varied across the five behaviors: Avoidance of evening screen/work exposure was reported by 11.4% of participants, compared with 87.4% reporting good sleep environment conditions, 76.0% avoiding daytime naps, 60.6% maintaining regular sleep schedules, and 49.1% avoiding afternoon caffeine. Higher adherence scores tended to accompany lower symptom scores. PwMS with higher adherence (≥3 good practices) had significantly lower fatigue impact (MFIS) and lower insomnia (AIS), sleepiness (ESS), and depression (HADS-D) scores than those with poor adherence (all *p* < 0.05). Anxiety (HADS-A) and FSS fatigue showed the same directional trend but did not reach significance (*p* > 0.05). Effect sizes for between-group differences were small to moderate, with the largest effect observed for the MFIS score. Table 2 shows symptoms for low- versus high-adherence groups.

### 2.3. Correlations Between Sleep Hygiene Adherence and Clinical Outcomes

We examined outcome scores across all six Sleep Hygiene Adherence Score levels (0–5) in Table 3. A generally decreasing pattern in symptom burden was observed with increasing levels of sleep hygiene adherence. This trend reached statistical significance for fatigue measures, including MFIS (Kruskal–Wallis H = 11.65, *p* = 0.020) and FSS (H = 10.13, *p* = 0.038). For other outcomes (AIS, ESS, HADS-A, HADS-D), a similar directional pattern was observed but did not reach statistical significance.

The relationship was not strictly monotonic. Participants with a score of 4 demonstrated marginally elevated mean scores in select outcomes when compared with those possessing a score of 3, which may reflect small subgroup sizes and clinical heterogeneity rather than a true reversal of the overall pattern.

Based on Spearman correlation analyses, the findings of the present study demonstrated that higher sleep hygiene adherence was associated with lower daytime sleepiness (ESS: r = −0.18, *p* = 0.020), lower anxiety (HADS-A: r = −0.16, *p* = 0.034), and lower depression (HADS-D: r = −0.15, *p* = 0.047). Correlations with MFIS, AIS, and FSS exhibited analogous trends, though these did not attain statistical significance.

### 2.4. Multivariate Analysis

Multivariate linear regression analyses were performed to evaluate whether sleep hygiene adherence was independently associated with symptom severity after adjustment for age, disability level (EDSS), and disease duration. Results of the multivariate regression analyses are presented in Table 4. Higher sleep hygiene adherence was independently associated with lower MFIS, ESS, HADS-A, and HADS-D scores, while associations with AIS and FSS did not reach statistical significance.

### 2.5. Gender-Stratified Analyses

Given the notable gender imbalance in the sample (74.9% female), gender-stratified multivariate regression analyses were conducted to explore the consistency of associations across genders. Sleep hygiene adherence scores did not differ significantly between females (mean 2.89 ± 1.00) and males (mean 2.73 ± 1.09; *p* = 0.40).

In females (*n* = 131), higher sleep hygiene adherence was independently associated with lower AIS scores (β = −0.86, 95% CI [−1.71, −0.01], *p* = 0.046) and lower ESS scores (β = −1.16, 95% CI [−2.00, −0.31], *p* = 0.008). In males (*n* = 44), higher sleep hygiene adherence was independently associated with lower MFIS scores (β = −4.96, 95% CI [−9.19, −0.73], *p* = 0.023) and lower HADS-A scores (β = −0.99, 95% CI [−1.93, −0.05], *p* = 0.040). Formal interaction tests were non-significant for all outcomes (all *p* > 0.20), suggesting no strong evidence for effect modification by gender.

## 3. Discussion

### 3.1. Principal Findings

The present study examined the association between sleep hygiene behaviors and symptom burden in a cohort of pwMS. The findings suggest that individuals with poorer sleep hygiene exhibited higher levels of fatigue severity, greater daytime sleepiness, and more pronounced symptoms of anxiety and depression. Notably, these associations remained significant after adjustment for confounding factors such as age, disease duration, and EDSS scores. Importantly, due to the cross-sectional design of this study, causal relationships cannot be established. It remains unclear whether better sleep hygiene contributes to reduced symptom burden or whether individuals with fewer symptoms are more likely to engage in healthier sleep behaviors. Reverse causation and bidirectional relationships are therefore plausible. This highlights the need for a personalized approach to sleep disorders in MS to optimize treatment. It is increasingly evident that behavioral and environmental factors play a crucial role in the quality of sleep [28,29,30]. In contrast to numerous disease-related factors, sleep hygiene is a potentially modifiable component of sleep health that can be addressed within the framework of routine clinical care [31,32]. In this study, pwMS who maintained regular sleep schedules, avoided daytime naps, and limited their evening caffeine and screen/work time, in addition to ensuring optimal sleep conditions, were associated with lower fatigue impact, fewer insomnia symptoms, and less excessive daytime sleepiness (EDS). Additionally, higher sleep hygiene adherence was related to lower depression levels. These results align with the evidence base linking healthy sleep behaviors to improved sleep quality and mood [8,9,33,34,35,36]. When sleep hygiene is considered a composite of multiple behaviors, a generally decreasing pattern of symptom burden with increasing adherence can be observed. This suggests a graded relationship between the number of health-promoting behaviors and clinical outcomes. This finding is consistent with the conceptualization of sleep hygiene as a multidimensional construct, in which individual behaviors represent relatively independent domains and may contribute additively rather than synergistically to sleep and related symptoms. Concurrently, this relationship is not exclusively linear, likely to reflect both the heterogeneity of the MS population and the behavioral nature of the construct, where different components may have varying relevance across individuals.

### 3.2. Sleep, Fatigue, and Mood Mechanisms

We observed an important trend towards reduced fatigue with better sleep hygiene, particularly on the MFIS scale, suggesting that secondary fatigue may be linked to improved sleep. Although the correlation with FSS was not statistically significant, lower MFIS scores in patients with good sleep hygiene may indicate a meaningful effect on functional fatigue. This supports Strober’s review, highlighting the role of sleep disturbance in MS-related fatigue. Strober found that sleep quality is a powerful, independent predictor of fatigue. It explains much of the variance in MFIS scores, regardless of a person’s level of disability or depression [37]. Fragmented or insufficient sleep may impair restorative processes, including glymphatic clearance and neuroimmune regulation, potentially contributing to fatigue and cognitive complaints [38,39,40,41].

Notably, the group with optimal sleep hygiene demonstrated significantly lower insomnia severity and EDS. This aligns with previous MS research reporting high insomnia rates and their impact on daily functioning. Specifically, the researchers identified female gender, anxiety, and bladder disorders as significant independent predictors of sleep disturbances in this population. These findings underscore the necessity of routine clinical screening for these comorbidities to facilitate early detection and the implementation of targeted interventions for insomnia [14]. Better sleep habits reduce nighttime arousal and fragmentation, thereby improving subjective sleep quality [42]. Irregular sleep schedules and evening stimulation may lead to circadian rhythm disruption and increased physiological arousal, promoting insomnia symptoms and reduced sleep efficiency [43]. EDS is frequently reported in MS and may result from both intrinsic disease mechanisms and insufficient or fragmented sleep. The data show that other sleep disorders fail to explain the rise in ESS values, implying that a separate set of mechanisms is responsible [44]. It is evident that behavioral factors, such as irregular sleep schedules, late evening stimulation, caffeine consumption, and inadequate sleep environments, may contribute to reduced sleep efficiency and insufficient restorative sleep. The persistence of associations following adjustment for EDSS suggests that these relationships are not solely explained by neurological disability.

The present study also demonstrated independent associations between sleep hygiene and symptoms of anxiety and depression. Better sleep hygiene was associated with lower depression and a non-significant trend toward lower anxiety. Poor sleep may also influence emotional regulation and stress reactivity, thereby contributing to anxiety and depressive symptoms [45,46,47]. This aligns with evidence that sleep and depression are interrelated; improving sleep can reduce depressive symptoms. In MS, depression is common and worsened by poor sleep [48,49,50]. Insufficient sleep has been found to be a factor in the occurrence of emotional dysregulation, a condition resulting from alterations in neuroendocrine and autonomic function, including, specifically, dysregulation of the hypothalamic–pituitary–adrenal axis [51]. Conversely, anxiety and depressive symptoms have been observed to have a negative effect on sleep behaviors and bedtime routines [52,53]. Thus, our findings suggest that promoting better sleep habits may also be associated with lower depressive symptoms. An improvement in motivation, the perception of fatigue, and the enhancement of the overall QoL may also be expected.

Anxiety and depression were assessed using the HADS scale, which does not capture other psychological dimensions, such as perceived stress [54]. Incorporating the Depression Anxiety Stress Scales (DASS-21) [55] in future studies could provide a more comprehensive view of the patient’s psychological landscape.

### 3.3. Gender Differences

The gender-stratified analyses conducted as part of this study indicated that there were broadly consistent associations between sleep hygiene and clinical outcomes, with no significant interaction effects. This finding suggests the presence of similar underlying patterns across genders. Despite the attainment of statistical significance in outcomes for both females and males, this is likely indicative of limited statistical power within the male subgroup, rather than the presence of true sex-specific effects. The present findings are consistent with those of recent studies, which indicate that, while women with MS more frequently report sleep disturbances, anxiety, and fatigue, men may present with a different symptom profile; however, the overall relationships between sleep, mood, and fatigue remain largely comparable across genders [14,56,57].

### 3.4. Evidence from Behavioral and Non-Pharmacological Interventions

Various behavioral and educational interventions, such as CBT-I, mindfulness, structured exercise, and targeted sleep education, have shown promising results in improving sleep quality and mitigating associated symptoms [58,59,60,61,62,63,64]. Specifically, CBT-I and mindfulness-integrated CBT have been shown to significantly improve sleep quality while simultaneously reducing fatigue and anxiety [63,64]. Physical activity interventions, such as the ACTIVE-FIT program, have further established a positive relationship between increased exercise and improved sleep outcomes in patients with relapsing–remitting MS [63]. Additionally, occupational therapy-based sleep programs and online meditation training have demonstrated efficacy in enhancing QoL and managing fatigue symptoms across the MS population [60,64].

### 3.5. Strengths and Limitations

This study has several limitations. The cross-sectional design of the study limits the ability to determine whether poor sleep hygiene contributes to increased symptom burden or whether patients with more severe symptoms experience greater difficulty in maintaining healthy sleep habits. Causal relationships cannot be inferred, and further longitudinal and interventional studies are required.

Sleep hygiene adherence was self-reported, and the Sleep Hygiene Adherence Score employed in this study constitutes a behavioral checklist based on commonly recommended sleep hygiene principles. It is important to note that the checklist has not yet undergone formal validation, and its psychometric properties remain to be formally established. This instrument was developed specifically for the current project because, at present, no standardized sleep hygiene scale has been formally validated within MS cohorts, and no pilot testing was conducted. Although internal consistency was low (Cronbach’s α = 0.18), this likely reflects the multidimensional nature of the construct. The measure should be interpreted as a composite index of distinct behaviors rather than a unidimensional scale. The binary scoring approach may not fully capture the complexity and variability of sleep-related behaviors. Furthermore, the dichotomous cutoff (≥3) employed for descriptive comparisons is somewhat arbitrary and has the potential to oversimplify a more complex, graded relationship. However, all primary analyses were conducted using the continuous score. Future research should employ validated instruments or formally validated tools adapted for MS populations.

While the ESS is a widely accepted measure of daytime sleepiness, it does not assess global sleep quality or specific nocturnal disturbances [65]. In individuals with chronic sleep impairment, an adaptation to sleep deprivation may lead to the underestimation of symptoms [66]. As our study focused on sleepiness rather than a multidimensional assessment of sleep, some aspects of sleep dysfunction may have been overlooked. Future research would benefit from incorporating the Pittsburgh Sleep Quality Index (PSQI) to provide a more sensitive and comprehensive evaluation of sleep health in this patient group [67]. 

Furthermore, the demographic and clinical profile of our sample may limit the generalizability of these findings across the full spectrum of MS. Our inclusion criteria were restricted to individuals with an EDSS < 7.0, and the cohort’s relatively low median EDSS score indicates a population with predominantly mild-to-moderate functional impairment [68]. Future research should include more diverse cohorts, including those with higher disability scores, to determine if these associations hold true in more advanced stages of MS.

Beyond functional disability, another factor not captured in our analysis was participants’ physical activity levels, which may serve as a significant confounding factor. Physical activity is known to have a bidirectional relationship with sleep quality, and it remains a key non-pharmacological intervention for managing MS-related fatigue and mood disturbances [69,70]. Future research should integrate objective or standardized subjective measures of physical activity to clarify how movement patterns interact with sleep in this population.

The predominance of female participants reflects the epidemiology of MS but may limit the generalizability. Gender differences in sleep regulation, mood disorders, and fatigue have been well documented and may influence the observed associations. The differences in statistically significant outcomes between females and males should be interpreted with caution and may reflect limited statistical power in the male subgroup rather than true sex-specific effects. Future studies should explore potential gender-related differences in sleep hygiene and symptom burden in MS.

Although key clinical variables (age, EDSS, and disease duration) were included in the regression models, other potentially important confounders, such as chronotype, physical activity, total daily screen time, exact quantity and timing of caffeine or alcohol consumption, occupational factors (e.g., shift work), and psychosocial stress, were not assessed. These factors may influence both sleep hygiene behaviors and clinical outcomes and could partially account for the observed associations. Future studies should incorporate a broader range of covariates and perform sensitivity analyses.

Finally, the exclusive reliance on self-report instruments is a limitation, as it introduces the risk of shared method bias and social desirability effects [71]. In sleep research, a discrepancy often exists between subjective reports and objective sleep parameters (e.g., as measured by actigraphy or polysomnography), particularly in populations with chronic illness where ‘sleep state misperception’ may occur [72,73]. Without objective assessment, it remains unclear whether improved sleep hygiene corresponds to physiological changes in sleep architecture versus improvements in the patient’s subjective appraisal of their symptoms. Future research should prioritize the inclusion of objective monitoring tools to provide a more robust and multi-dimensional validation of these behavioral associations.

Despite these limitations, the study has several strengths. To the best of our knowledge, this is one of the first studies specifically examining behavioral sleep hygiene in pwMS using a structured checklist approach. The study comprised a moderately large and clinically well-characterized cohort of patients. A variety of clinical scales with established validation were utilized to evaluate symptoms of fatigue, insomnia, EDS, anxiety, and depression. Furthermore, multivariate regression models were applied to adjust for important clinical confounders, including age, disease duration, and disability level (EDSS). Future research should use longitudinal, prospective interventions to confirm whether improving sleep hygiene alleviates MS symptoms.

### 3.6. Implications for Clinical Practice

The findings of this study may have the potential to inform clinical practice. The evaluation of sleep hygiene demands minimal duration and can be easily integrated into routine neurological practice. The identification of maladaptive sleep behaviors may assist clinicians in recognizing potentially modifiable contributors to fatigue and psychological symptoms in pwMS. In clinical settings, sleep hygiene assessment should be integrated into routine MS care using pragmatic screening tools like our five-item checklist. As sleep behaviors appear independent of neurological disability (EDSS), targeted behavioral counseling offers a cost-effective, non-pharmacological approach to alleviating fatigue and depressive symptoms. These interventions represent a valuable adjunct to established treatments like CBT-I, facilitating a more comprehensive approach to symptom management in pwMS.

## 4. Materials and Methods

A total of 175 patients with clinically confirmed MS were included in the study. Diagnoses were established according to the McDonald criteria from 2010 and 2017 [74,75]. Participants were adults aged 18–65 years with an EDSS score of <7.0 [68,76]. All pwMS were diagnosed and treated at the Neurology Outpatient Clinic of the Department of Neurology, University Clinical Centre, Medical University of Silesia in Katowice, Poland. The study was conducted between July 2020 and August 2023.

**Figure 1:**
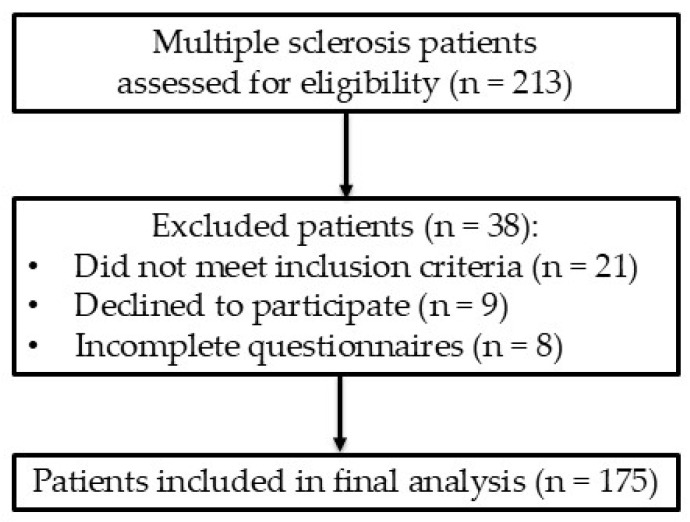
Flowchart of study participant recruitment.

The study protocol was reviewed and approved by the Bioethics Committee of the Medical University of Silesia in accordance with the Declaration of Helsinki (PCN/0022/KB1/46/20). All participants were informed of the study’s objectives and provided written informed consent before inclusion. Exclusion criteria encompassed conditions that could influence sleep quality, such as respiratory diseases, heart failure, previously diagnosed mental disorders, and the abuse of alcohol or hypnotic medications. A flowchart of participant recruitment for this study is presented in Figure 1.

Patients completed self-report questionnaires. The dataset comprised demographics and responses to sleep and symptom inventories. The variables described below were identified as being of particular significance for the present study.

### 4.1. Sleep Hygiene Assessment

The assessment of sleep hygiene practices was conducted using a brief, study-specific, five-item checklist that was developed for the specific purposes of this project. As no standardized sleep hygiene instrument has been specifically validated in MS populations, a study-specific checklist was constructed based on established sleep hygiene recommendations. This checklist was based on core recommendations from the American Academy of Sleep Medicine (AASM) and the National Sleep Foundation (NSF) [77,78]. The items captured key behavioral domains consistently associated with sleep quality in prior literature:

(1) Maintaining a regular sleep–wake schedule;
(2) Avoiding daytime naps;
(3) Avoiding caffeine or energy drinks in the afternoon or evening;
(4) Limiting stimulating activities in the evening (screen use or work);
(5) Ensuring an appropriate sleeping environment (quiet, dark, bedroom used primarily for sleep).

The scoring of each item was conducted dichotomously (yes/no) and subsequently coded as 1 (sleep-promoting behavior present) or 0 (absent). The items were then summed to yield the Sleep Hygiene Adherence Score (range 0–5), with higher scores indicating better adherence to sleep hygiene recommendations (Supplementary Materials, Table S1). For description, participants were classified into two categories according to their adherence to sleep hygiene practices: those with lower adherence (score 0–2) and those with higher adherence (score ≥ 3). The cutoff value was selected to reflect adherence to most recommended behaviors. The dichotomous grouping was employed for descriptive purposes only, while all primary inferential analyses were conducted using the continuous variable. The sleep hygiene checklist is a pragmatic behavioral measure that has not yet undergone formal psychometric validation. The internal consistency of the checklist was low (Cronbach’s α = 0.18). It is important to note that the Sleep Hygiene Adherence Score is conceptualized as a formative composite index rather than a reflective psychometric scale. Each item represents a distinct, independently actionable sleep behavior, and items are not expected to covary. Consequently, Cronbach’s α = 0.18 assumes limited interpretability in this context due to the presence of item intercorrelations. The inter-item Spearman correlations ranged from r = 0.01 to r = 0.12 (all *p* > 0.10), indicating behavioral independence across domains (Supplementary Materials, Table S2).

### 4.2. Fatigue

Fatigue was assessed using two validated tools for MS patients: the Fatigue Severity Scale (FSS) and the Modified Fatigue Impact Scale (MFIS). The FSS consists of nine items measuring fatigue severity, with scores ≥ 36 indicating clinically significant fatigue [79]. The MFIS includes 21 items assessing the impact of fatigue in three domains: physical (9 items), cognitive (10 items), and psychosocial (2 items). Scores > 38 reflect a substantial effect on daily functioning and QoL [80]. The MFIS has been adapted and validated for the Polish population [81].

### 4.3. Insomnia

The severity of insomnia was measured using the Athens Insomnia Scale (AIS), an eight-item self-administered tool based on the 10th revision of the International Statistical Classification of Diseases and Related Health Problems (ICD-10) diagnostic criteria for insomnia. It has been validated in the Polish population (Cronbach’s α = 0.89) [82,83]. A score ≥ 6 indicates borderline insomnia, while a score > 10 signifies insomnia. The total score ranges from 0 to 24.

### 4.4. Daytime Sleepiness

The Epworth Sleepiness Scale (ESS) is an eight-item questionnaire assessing subjective sleepiness [84], reflecting the likelihood of falling asleep during daily activities. Scores > 10 indicate excessive daytime sleepiness, and scores > 14 indicate pathological sleepiness.

### 4.5. Mood Disorders

The Hospital Anxiety and Depression Scale (HADS) is a 14-item self-report tool assessing anxiety (HADS-A) and depression (HADS-D) in somatically ill patients [85]. We used the validated Polish version [86]. For each subscale, scores ≥ 8 indicate borderline cases, while scores > 11 indicate definite cases. This instrument was specifically selected due to its focus on non-somatic symptoms, making it a robust choice for MS cohorts where physical symptoms like fatigue and sleep–wake disturbances could confound the assessment of anxiety and depression [87].

### 4.6. Statistical Analysis

Descriptive statistics were used to summarize the characteristics of the sample. Quantitative variables were reported as the mean ± standard deviation (SD) and qualitative variables as counts and percentages. Normality was assessed using the Shapiro–Wilk test. As the distributions were not normal, intergroup comparisons of quantitative variables were performed using the Mann–Whitney U test. Sleep hygiene adherence was quantified using a study-specific five-item behavioral index developed for this project described above. Participants were divided into two groups based on their adherence to sleep hygiene practices: those with low adherence (score of ≤2) and those with high adherence (score of ≥3). Inter-item Spearman correlations were computed to characterize the independence of the five sleep hygiene items. To investigate the existence of a relationship between adherence to sleep hygiene measures and clinical outcomes, the mean values for each outcome variable were calculated across all levels of the Sleep Hygiene Adherence Score (0–5). The assessment of differences across levels was conducted by means of the Kruskal–Wallis test. Its association with symptom severity was examined by correlating total scores with standardized measures (FSS, MFIS, AIS, ESS, and HADS). Associations between sleep hygiene adherence and symptom severity were evaluated using Spearman correlation coefficients. Multivariate linear regression models were constructed to evaluate the independent association of sleep hygiene adherence with symptom severity. The models were adjusted for age, disability level (EDSS), and disease duration. Consequently, separate models were constructed for MFIS, AIS, ESS, FSS, HADS-A, and HADS-D. Analyses stratified by gender were performed to explore potential gender differences. Furthermore, interaction terms (gender × sleep hygiene adherence) were included in the regression models to assess potential effect modification. Multivariate linear regression models are presented with beta coefficients, 95% confidence intervals, and *p*-values. The statistical significance was set at α = 0.05. Analyses were performed in R v3.6.2 (R Core Team, Vienna, Austria, 2019).

## 5. Conclusions

This analysis of an MS cohort demonstrated that better adherence to sleep hygiene is associated with lower fatigue impact, fewer insomnia complaints, lower daytime sleepiness, and milder depressive symptoms. Gender-stratified analyses showed consistent associations in both females and males, though the pattern of significant outcomes differed by gender. Despite the cross-sectional design’s inability to draw causal conclusions, the findings suggest that sleep hygiene may represent a clinically relevant and potentially modifiable factor contributing to the symptom burden experienced by pwMS. The assessment and improvement of sleep hygiene may represent a simple and accessible component of comprehensive MS care. Incorporating a brief sleep hygiene assessment into routine care may support a more comprehensive approach to symptom management. Further longitudinal and interventional studies are required to determine whether improving sleep hygiene leads to clinically meaningful improvements in pwMS.

## Figures and Tables

**Table 1:** Demographic and clinical characteristics of the study group of patients with multiple sclerosis.

Characteristic	Value (*n* = 175)
Demographics	
Age [years], mean ± SD	38.8 ± 11.2
Male sex, *n* (%)	44 (25.1%)
Female sex, *n* (%)	131 (74.9%)
BMI, kg/m^2^, mean ± SD	24.4 ± 4.6
Clinical features	
MS subtype, *n* (%)	
RRMS	157 (89.7%)
SPMS	6 (3.4%)
PPMS	12 (6.9%)
Disease duration, *n* (%)	
<1 year	22 (12.6%)
1–5 years	61 (34.9%)
6–15 years	69 (39.4%)
>15 years	23 (13.1%)
EDSS median (range)	2.0 (0–6.5)
Treatment status, *n* (%)	
Currently on DMT	165 (94.3%)
No DMT	10 (5.7%)
Socioeconomic factors, *n* (%)	
Higher education	84 (48.0%)
Smoking	31 (17.7%)

Note: BMI—body mass index; DMT—disease-modifying treatment; EDSS—expanded disability status scale; MS—multiple sclerosis; PPMS—primary progressive multiple sclerosis; RRMS—relapsing–remitting multiple sclerosis; SD, standard deviation; SPMS—secondary progressive multiple sclerosis.

**Table 2:** Mean (±SD) symptom scores in patients with multiple sclerosis with low versus high adherence in the Sleep Hygiene Adherence Score, with effect sizes (Cohen’s d).

Outcome	Low Adherence Sleep Hygiene (Mean ± SD)	High Adherence Sleep Hygiene (Mean ± SD)	*p*-Value	Effect Size
FSS sum (fatigue)	29.3 ± 14.8	25.3 ± 13.3	0.072	0.28
MFIS total (fatigue)	35.2 ± 21.7	26.7 ± 19.7	**0.010**	**0.41**
AIS sum (insomnia)	7.1 ± 4.8	5.6 ± 4.5	**0.048**	**0.32**
ESS sum (sleepiness)	6.5 ± 5.3	4.7 ± 4.3	**0.017**	**0.37**
HADS-A	6.8 ± 3.9	5.9 ± 3.8	0.121	0.23
HADS-D	4.8 ± 4.2	3.6 ± 3.5	**0.040**	**0.31**

Note: Bold *p* < 0.05. AIS, Athens Insomnia Scale; ESS, Epworth Sleepiness Scale; FSS, Fatigue Severity Scale; HADS-A, Hospital Anxiety and Depression Scale—Anxiety Subscale; HADS-D, Hospital Anxiety and Depression Scale—Depression Subscale; MFIS, Modified Fatigue Impact Scale; SD, standard deviation.

**Table 3:** Clinical outcomes by increasing levels of sleep hygiene adherence score in patients with multiple sclerosis.

Sleep Hygiene Adherence Score (Points)	*n* (%)	MFIS	AIS	ESS	FSS	HADS-A	HADS-D
0	2 (1.1%)	54.5 ± 31.8	9.5 ± 7.8	9.5 ± 10.6	45.5 ± 24.7	10.0 ± 5.7	10.0 ± 5.7
1	15 (8.6%)	34.3 ± 23.1	7.8 ± 5.0	7.8 ± 5.3	32.5 ± 15.2	6.5 ± 4.9	5.2 ± 4.0
2	43 (24.6%)	34.6 ± 21.1	6.7 ± 4.7	5.9 ± 5.0	27.4 ± 14.1	6.7 ± 3.5	4.4 ± 4.1
3	69 (39.4%)	24.0 ± 19.9	5.4 ± 4.2	4.6 ± 4.3	23.0 ± 12.3	6.1 ± 4.0	3.4 ± 3.4
4	40 (22.9%)	32.7 ± 18.7	5.7 ± 4.9	5.2 ± 4.5	30.0 ± 14.5	5.9 ± 3.5	4.0 ± 3.8
5	6 (3.4%)	19.2 ± 16.2	7.7 ± 4.8	4.0 ± 2.1	20.0 ± 9.4	2.8 ± 1.8	3.0 ± 1.4
Kruskal–Wallis *p*-value		**0.020**	0.184	0.198	**0.038**	0.100	0.427

Note: Values are presented as mean ± SD. Differences across levels were assessed using the Kruskal–Wallis test. Bold *p* < 0.05. AIS, Athens Insomnia Scale; ESS, Epworth Sleepiness Scale; FSS, Fatigue Severity Scale; HADS-A, Hospital Anxiety and Depression Scale—Anxiety Subscale; HADS-D, Hospital Anxiety and Depression Scale—Depression Subscale; MFIS, Modified Fatigue Impact Scale.

**Table 4:** Multivariate linear regression analysis of the association between sleep hygiene adherence and clinical outcomes in patients with multiple sclerosis.

Outcome	β	95% CI	*p*-Value
MFIS	−3.24	−6.06; −0.41	**0.025**
AIS	−0.62	−1.29; 0.06	0.072
ESS	−0.85	−1.54; −0.16	**0.016**
FSS	−1.60	−3.60; 0.40	0.116
HADS-A	−0.67	−1.23; −0.11	**0.019**
HADS-D	−0.62	−1.17; −0.08	**0.026**

Note: Models were adjusted for age, EDSS, and disease duration. Bold *p* < 0.05. AIS, Athens Insomnia Scale; ESS, Epworth Sleepiness Scale; FSS, Fatigue Severity Scale; HADS-A, Hospital Anxiety and Depression Scale—Anxiety Subscale; HADS-D, Hospital Anxiety and Depression Scale—Depression Subscale; MFIS, Modified Fatigue Impact Scale; β—unstandardized regression coefficient; CI—confidence interval.

## Data Availability

The data presented in this study are available from the corresponding author upon request. The data are not publicly available due to legal restrictions [any type of medical data includes sensitive information].

## Supplementary Materials

The following supporting information can be downloaded at: https://www.mdpi.com/article/10.3390/clockssleep8020024/s1, Table S1: Sleep Hygiene Adherence Score. Table S2: Inter-item Spearman correlations for the Sleep Hygiene Adherence Score.

## Abbreviations

The following abbreviations are used in this manuscript:

MS	Multiple Sclerosis
CBT-I	Cognitive Behavioral Therapy for Insomnia
EDSS	Expanded Disability Status Scale
FSS	Fatigue Severity Scale
MFIS	Modified Fatigue Impact Scale
AIS	Athens Insomnia Scale
ICD-10	10th revision of the International Statistical Classification of Diseases and Related Health Problems
ESS	Epworth Sleepiness Scale
HADS	Hospital Anxiety and Depression Scale
BMI	Body Mass Index
RRMS	Relapsing–Remitting Multiple Sclerosis
SPMS	Secondary Progressive Multiple Sclerosis
PPMS	Primary Progressive Multiple Sclerosis
