# Supplementary material for: Sleep Hygiene and Symptom Burden in Multiple Sclerosis: A Cross-Sectional Study
Source: Clocks Sleep. 2026 Apr 30;8(2):24. doi: 10.3390/clockssleep8020024 (PMC13214699; doi:10.3390/clockssleep8020024)
Supplement: Supplementary file 1 [file clockssleep-08-00024-s001.zip › clockssleep-4223761-supplementary.pdf]

Supplementary Materials:

Table S1. Sleep Hygiene Adherence Score.

Sleep hygiene questions - Sleep Hygiene Adherence Score
1. Do you maintain regular sleep and wake times?
□ No
□ Yes
2. Do you take afternoon naps?
□ No
□ Yes
3. Do you consume coffee or energy drinks in the afternoon?
□ No
□ Yes
4. Do you engage in stimulating activities in the evening (e.g., work-related tasks, TV watching, internet use)?
□ No
□ Yes
5. Do you have adequate sleeping conditions (a quiet, dark bedroom used exclusively for sleeping)?
□ No
□ Yes

Table S2. Inter-item Spearman correlations for the Sleep Hygiene Adherence Score.

	**Regular sleep schedule**	**No daytime naps**	**No evening caffeine**	**No evening screen/work**	**Optimal sleep environment**
**Regular sleep schedule**	—	0.12	0.12	0.03	0.01
**No daytime naps**	0.12	—	0.12	−0.09	0.03
**No evening caffeine**	0.12	0.12	—	0.11	−0.11
**No evening screen/work**	0.03	−0.09	0.11	—	−0.03
**Optimal sleep environment**	0.01	0.03	−0.11	−0.03	—

Note: Spearman correlation coefficients between individual items of the Sleep Hygiene Adherence Score. All correlations were non-significant (all $p > 0.10$; $n = 175$).
